# Adenoids in Relation to Structural Changes

**Published:** 1902-06

**Authors:** F. Park Lewis

**Affiliations:** Buffalo, N. Y.


### International Dental Journal.

Vol. XXIII. June, 1902. No. 6.

### Original Communications.[1]

### ADENOIDS IN RELATION TO STRUCTURAL CHANGES.[2]

BY F. PARK LEWIS, M.D., BUFFALO, N. Y.

You will pardon me if I begin my paper to-night with a few fundamental propositions which are doubtless known to every one of you, in order to make more clear and consecutive subsequent details which may not be so well known.

By adenoids we understand an hypertrophied condition of the lymphoid tissues, normally found in the vault of the pharynx, posterior to the nasal openings. In structure these tissues form a portion of what is known as the tonsillar ring, composed of the faucial tonsils on either side, the lingual tonsils, consisting of a number of enlargements at the base of the tongue, and the pharyngeal tonsil in the vault of the pharynx. In a normal condition this last, the pharyngeal, or, as it is sometimes called, Luschka's tonsil, consists of a number of closed lymph-follicles in the mucous membrane, surrounded by loose-meshed connective tissue. There

[1] The editor and publishers are not responsible for the views of authors of papers published in this department, nor for any claim to novelty, or otherwise, that may be made by them. No papers will be received for this department that have appeared in any other journal published in the country.

[2] Read before The New York Institute of Stomatology, February 12, 1902.

is a soft variety of what is still called adenoids, which is predominantly an enlargement of this mucous tissue; when the connective tissue is in excess it is quite another thing. It has been deplored that either should be called adenoids, for not even the latter, or true adenoid, is an hypertrophy of the lymph-gland. Practically, it might almost as well be, however, for the contraction and hardening of the surrounding tissue cuts off the lymph flow as completely as if it were the lymph-vessels themselves that were affected.

It must be remembered that the lymphatic tract is the channel through which the nutritive elements of the blood are carried to their destination. The lymph in the human body is its life. It is the food of the cell. Upon its free flow is dependent the functional activity and the normal development of every tissue.

The lymphatic glands in the vault of the pharynx are part of a chain extending through the openings of the skull, carrying nutrition directly to the brain itself. Immediately above this region is the sella turcica, which contains the pituitary body. Those of you who have been following modern medical research will remember that in the strange and interesting condition called acromegalia, or gigantism, in which abnormal enlargement of the whole bony structure occurs, post-mortem investigations have discovered an enlargement of this pituitary body as a most constant feature.

The pituitary body is composed of two lobes, one of which is true brain tissue, and the other a lymphatic gland. The chain is continuous from the lymphoid tissue in the vault of the pharynx, by way of the arterial seats, to the substance of this gland itself.

Now, when we realize how profound is the disturbance of nutrition throughout the entire system, when the functions of the pituitary body are disturbed, it may be readily understood that any obstruction to the free flow of lymph through glands so nearly adjacent as those of the pharynx might seriously interfere in other ways with the processes of development.

A disturbance of function always precedes a change of structure; and if it can be shown that functional disturbances are produced in adjacent organs by hypertrophies of the lymphoid tissues in the pharynx and the post-nasal space, the conclusion is justified that in case the hypertrophies are allowed to continue, structural changes must follow.

Should these hypertrophies occur during the period of development in children, we are warranted in making the tentative deduction that where functional disturbances have been observed, interferences with development are likely to occur, as structural changes would in an adult.

Now, clinically the relationship between adenoids and deformities of the face, nose, and chest have been so constantly observed that the facial appearance has come to be accepted as a characteristic indication of the presence of adenoid vegetations in the nasopharynx.

Their relationship to some forms of ear disease, while often recognized, has not yet received the attention which its importance demands, while the connection of hypertrophies of this nature with the eye, and its development, has scarcely received any consideration whatsoever. Indeed, I cannot find that this aspect of the subject has been studied at all.

The typical adenoid face is well known. The enlargement of tissue obstructing the posterior nares so narrows the caliber of these openings that breathing is possible only with the mouth partially open. The upper lip is commonly shortened, the lower lip droops. This not only gives a stupid expression to the face, but the mental processes are actually slow. The child finds difficulty in concentrating the attention on any subject, and reflex symptoms of the most varied character are not uncommon. (Among these may be mentioned the night terrors from which children suffer, wetting the bed, persistent headaches not relieved by ordinary measures, marked catarrhal symptoms, etc.)

The form of skull which is found so commonly in cases of this character is that in which the upper jaw—sometimes, indeed, the lower as well—is narrowed laterally. The face is compressed from side to side, the *bony palate* forms a high arch. The teeth are so crowded that they find no room for regular eruption, and to the unpleasant rat-shaped face is added, therefore, the disfigurement of an ill-shaped mouth with irregular teeth. The high arch constituting the bones of the roof of the mouth presses up the septum of the nose, causing it to deviate, and laying the foundation for future obstructions in the nostril. In a word, the mechanical fact of crowding the face together throws all the bony structures out of their true relationships, and is the foundation of disturbances of a most serious character.

Much thought has been given to the manner in which this peculiar conformation occurs, and the conclusion seems to have been that the obstruction of the nose by the adenoids, necessitating mouth-breathing, has gradually drawn up the, as yet, plastic structure of the roof of the mouth, until this conformation has resulted. That this is not a complete explanation, although it may be a contributing element, is shown by two facts: first, that the condition is sometimes present at so early an age that it could not have been produced by suction, as this process would necessitate time, and the condition must have been congenital; and secondly, because it has been exhibited in branches of families to such an extent that the element of heredity must enter largely into it.[1]

A case that came under my observation recently was that of twin children, in one of whom this facial distortion was conspicuous, while in the other the face was broad and the jaws roomy. The first child was a mouth-breather from birth. The mother assured me that this child was the elder, and had been carried low in the pelvis, leading to the possible inference that the weight of the other child, by continued pressure, might have changed the shape of the skull. The facial conformation characteristic of this condition frequently is found in a mother and her whole family of children. It would be interesting to know whether a narrow pelvis might not sometimes be instrumental in producing the narrow head, which is the foundation of the whole condition.

But while all this affords a fascinating field for speculation and theory, it is not essential that we should know *how* it is produced, it is sufficient to realize that a condition which will so crowd the jaws that the teeth have not sufficient room for their eruption, may also so impinge upon the lymph-channels as to produce strangulation of these structures, with subsequent hypernutrition and the abnormal development of connective tissues, which we call adenoids.

Therefore, although the consensus of opinion seems to have been that suctional breathing, the result of adenoids, has been the cause of the narrowed face and head, with all the attendant symptoms which we have been considering, I cannot but believe it more probable that the reverse is the fact,—that the narrow head and face, having been produced by any means whatsoever, would

---

[1] That congenital adenoids are not infrequent is shown by Leopold Chaureau, who reports having made fifty operations on new-born infants. Gaz. hebdom. de méd. et de chir., April 8, 1900.

naturally result in obstruction of lymph-passages, with all the varied and unfortunate phenomena which such obstruction implies.

(I should say in parentheses, that the narrow face with its attendant obstructions is not the only thing that could crowd upon the delicate developing tissues until adenoid growths result. But cases where abnormalities of the face are not present would be the concern of the general physician, not of the dental surgeon, and therefore will not be considered in this paper.)

If my theory is correct its importance to the dental profession is obvious.

By an early expansion of the jaw, not only is the unpleasant rat-mouth moulded into better form and the teeth given room to develop properly, the bony arch lowered and the lips thus brought into place, the nasal septum straightened and the features given a dignity and the mouth an efficiency that they would otherwise lack, but the lymph-channels are unstopped. The first of these are chiefly mechanical results, the latter is concerned with the essential elements of structural nutrition.

The different organs that may be affected when nutrition is interfered with, and the various ways in which deviations from the normal occur, interest chiefly, of course, the general physician. But the whole subject, even pushed to somewhat wearisome detail, is of importance to the dental surgeon, as it urges upon him the necessity of recognizing the less striking of these cases.

Then, when recognized, in instances where the facial deformities are not extreme, or but slightly apparent, there is sometimes difficulty in persuading the parents to submit their children to the measures necessary to produce orthodontia. The process is a long and expensive one, and entails not a little discomfort, and the parent is likely to urge postponement, hoping that the necessity for the operation may disappear with growth. A knowledge of the remote and serious difficulties which may be the outcome of this condition will afford the surgeon in the several details so many weapons for the defence of his position.

One of the most obvious effects of adenoid obstructions in the vault of the pharynx is the mechanical interference with the ventilation of the middle ear through the Eustachian tubes. According to the location of the adenoid growths, either one or both sides may be involved. The necessity of an unimpeded passage through the nose, as related to the ear, may be immediately demonstrated, in a normal condition, by the simple experiment of closing the nostrils. This may be made more clear by at the same time trying to swallow. We find this same process emphasized by a common cold in the head, which gives the sensation known as a stuffy feeling in the head and ears.

Now, with enlarged tissues in the throat it requires a very slight inflammation to so interfere with the mouths of the Eustachian tubes as to almost completely shut off the entrance of air. The continuity of tissue readily allows an extension of the inflammation to the middle ear; the drum membrane also becomes inflamed. The swollen tissues of the tympanus ultimately fill with pus, and the pain is excruciating until the membrane is ruptured or an opening artificially made by the surgeon's knife, and we have all the classic symptoms of otitis, with the possible resultant conditions of a chronic otorrhœa, mastoid involvement, cerebral abscess, sinus thrombosis, or even general septicæmia.[1]

I think it rarely happens, when children have repeated attacks of earache, that investigation will not show the presence of adenoids.

The continued obstruction of the Eustachian tubes without inflammatory complications develops a progressive form of catarrhal deafness, for which no relief can be obtained so long as the obstructions continue.

This is a kind of chronic deafness, the origin of which is frequently overlooked. In the course of time organic changes occur in the structures of the middle ear, such as retraction and thickening of the drum membrane, adhesions at the joints of the ossiculæ, and, in some cases, involvements of the nervous structures of the inner ear itself, with permanent narrowing of the Eustachian tube. It is, of course, then too late for the removal of the cause to produce beneficial results. With a knowledge of these facts it is obvious that no case of deafness in a child should ever be allowed to continue without a thorough examination of the throat to determine whether obstructions are present or not.

The large number of deaf mutes in whom adenoid vegetations are found to exist would seem to justify the conclusion that these

---

[1] A. Plotier found twenty cases of adenoids in thirty-eight cases of death from diphtheria. Laryngoscope, August, 1899.

obstructions were either congenital or prenatal, and that deafness being present at so early a period allowed the unused ear structures to degenerate, while the brain centres for audition remained undeveloped.

The remarkable experiments of Urbanschitsch in the training of mutes with ear-trumpets would indicate that in many of these cases some degree of hearing might be secured by training the ear, if the auditory passages are free from obstructions.

Of course, it is only before structural changes have taken place that beneficial results may be anticipated.

Structural changes take place in a comparatively short time, when, added to disuse, we have a choking of the channels of nutrition. The presence of adenoids in the throat may, therefore, be responsible for structural changes in the ear, and not many years be required in the process.

This makes evident the necessity for careful diagnosis and prompt care of young children when these conditions may exist.

It is an accepted idea that adenoids left to themselves will atrophy at adolescence, but this is only partially true. The growth may, and probably does, shrink somewhat, but when connective tissue changes have once occurred, the adenoid persists, although the enlargement of the vault of the pharynx coincident with normal growth lifts the enlarged tissue so that it no longer obviously obstructs the nares, and while it may be producing disastrous changes its presence is often totally unsuspected.

I have myself removed large adenoids from a man thirty-five years of age, very greatly relieving his deafness in one ear. In the other tissue changes had occurred to such a degree that improvement of hearing was impossible.

It is the persistence of these unrecognized progressive tissue changes that lays the foundation for much of the incurable deafness of adult life. And when these are the result of abnormal growths, which may in turn be the result of abnormal mouth development, not only must the growths be removed, but the care of the jaws and teeth must not be too long postponed.

Now, I have reason to believe that the obstruction of the flow of lymph carrying nutrient elements to the ear has possibly as much to do with the results as the mechanical obstruction in shutting off the Eustachian tubes. But upon this subject the final word has by no means yet been said.

When we speak of the eye, the dental relationship would seem to be less apparent, but, as a matter of fact, we find here similar conditions, giving rise to equally marked results.

And if my theory is correct, that distorted facial development produces adenoidal growths, and that adenoids are responsible for various eye troubles not hitherto traceable to any direct cause, the relationship is discovered to be intimate.

If, as was shown in regard to the ear, functional difficulties arise in conjunction with adenoids and disappear on their removal, and we remember that structural changes invariably follow a persistent aberration of function, we may say that here too, similar conclusions follow like premises. That is to say, structural changes in the eye may occur as the result of lymphoid hypertrophies.

And furthermore, if these obstructions to normal nutrition occur during the plastic period before development is complete, they may interfere with the normal growth of the organ, and arrested development be the result.

No one, two, or three cases would be sufficient to prove the relationships of adenoids to disturbances of the eyes, but when over and over again cases that have evaded all ordinary medical or surgical measures have been immediately relieved by the removal of lymphoid growths, the causative relationship would seem to be demonstrated.

One of the commonest conditions which I have observed in this connection is that in which the conjunctiva is congested, the eyes weak and suffused, a condition for which local measures have proved in many instances totally insufficient, but which was at once relieved by the discovery and removal of adenoids.[1]

A peculiarly interesting case came under my observation recently of a child having a persistent overflow of tears from one eye. The lachrymal duct was found perfectly normal, and the overflow, which had for several years resisted treatment, ceased within a week after removal of a small mass of adenoids that had been too slight to occasion the child any other discomfort.

Various weaknesses of the ocular muscles, for which suitable glasses and even operative measures have been unavailing, have

---

[1] Coppez and Snellen have both recorded frequent instances in which follicular conjunctivitis is associated with post-nasal adenoids. Archiv. d'Ophthalmol., January, 1899.

regained their normal balance after the nasopharynx has been cleared.

Lauren's case, in which strabismus disappeared after an operation for adenoids, and Miles's cases of æsthenopia, which subsided after similar operations, lead directly, as do all of these cases, to a consideration of the development of the eye and the causes which may modify it.[1]

At birth the eye of a child is in the same undeveloped conditions as are the other organs. As the child grows the eye develops, enlarges, and becomes functionally able to do the work which is required of it. This process of development extends normally through the growing period of the child's life. If for any reason it should be interrupted, the eye remains throughout life that of the child. It is then too flat, never having reached a full contour, and is called a hypermetropic, or over-sighted, eye. Such eyes are peculiarly prone to convergence, and are apt to be dull-sighted.

A laryngologist by the name of Ziem has recently made some very interesting experiments, that are most suggestive in this connection. He sewed up the nostrils of young rabbits, and found that on the side from which air was excluded the eye remained in a permanently undeveloped condition. In other words, it remained a flat or hypermetropic eye, while that on the opposite side reached its normal development.

Now, in my own experience such a large number of these hypermetropic and squinting eyes in children perhaps twelve years of age have been found in connection with lymphoid hypertrophies that it is impossible to believe the association accidental.

If it be true that the nutritive functions of the head are so profoundly impaired by nasopharyngeal hypertrophies, and if it be also true that these are again contingent upon corrigible deformities of the skull, I can only say again, if possible with still more emphasis, that as the responsibility in this class of cases often falls upon you, my friends of the dental profession, long before any developments occur which would bring them to the care of the general surgeon, the entire subject must be regarded as of an importance which can hardly be exaggerated.

---

[1] Thomas cites a case of a child of ten with divergent strabismus and mental disturbances following meningitis during infancy. After curettage of the nasal pharynx for adenoids the strabismus entirely disappeared. Presse Méd., October 29, 1898.

